# Gastrointestinal stromal tumors (GISTs) and synchronous intra-abdominal malignancies: case series of a single institution’s experience

**DOI:** 10.18632/oncotarget.27853

**Published:** 2020-12-29

**Authors:** Alexandros Diamantis, Athina A. Samara, Dimitrios Symeonidis, Ioannis Baloyiannis, Dionysia Vasdeki, Maria Tolia, Georgios Volakakis, Georgios Mavrovounis, Konstantinos Tepetes

**Affiliations:** ^1^Department of General Surgery, University Hospital of Larisa, Mezourlo, Larisa, Thessaly, Greece; ^2^Department of Radiotherapy/Radiation Oncology, Faculty of Medicine, School of Health Sciences, University of Crete, University Hospital of Heraklion, Crete, Greece; ^3^Faculty of Medicine, University of Thessaly, Mezourlo, Larissa, Greece

**Keywords:** gastrointestinal stromal tumor, mesenchymal tumors, synchronous malignancies

## Abstract

Background: Gastrointestinal stromal tumors (GISTs) quite often co-exist with other primary tumors, as seen in up to 33% of cases. In the literature such occurrences have primarily been described through case reports and rarely through case series, which is not sufficient to prove if there is an association between these two entities.

Materials and Methods: We conducted a retrospective study using medical and pathological records from sixty-nine patients who underwent surgical treatment for GIST in a single university surgical department between 2011 and 2019. Seven cases of GIST accompanying a synchronous primary tumor were identified and included in the study.

Results: Survival analysis comparing the overall survival of patients with single GIST versus patients with concurrent GIST and another primary tumor, has shown no statistically significant difference between these two groups (*p* = 0.19). However, when comparing the recurrence rate, patients with synchronous GISTs and another primary tumor have a statistically significant increased possibility for recurrence (*p* = 0.02). Statistical analysis comparing the size of GISTs between the two groups has shown that patients with single GIST have larger tumors than patients with synchronous tumors (*p* = 0.048).

Conclusions: The synchronous occurrence of GISTs and other intra-abdominal tumors is more common than previously considered, though it is not yet clear if there is a causal association for the concomitant occurrence. Further studies are required to elucidate the genetic and molecular mechanisms of carcinogenesis and progression associating GIST and synchronous tumors.

## INTRODUCTION

Although they are rare neoplasms, gastrointestinal stromal tumors (GISTs) represent the most common mesenchymal tumors of the gastrointestinal (GI) tract. They originate from the cells of Cajal, which are responsible for autonomous GI movement [1–3]. GIST incidence ranges between 0.1–1% of all gastrointestinal malignancies, occurring in adults 55–65 years of age [2–4]. The majority manifest as sporadic solitary lesions; nonetheless, they may also develop in a familial fashion such as in neurofibromatosis and Carney triad [5]. The most common localization of GISTs is the stomach (50–60%), followed by the small intestine (30–40%), the colon-rectum (5–10%) and rarely the oesophagus (< 5%) [3, 6]. Less frequently, GISTs may develop in extra-GI sites, mainly the mesentery, the omentum and the retroperitoneum.

With a wide spectrum of presentation, GISTs can range from small and benign nodules that are incidentally detected, to frankly malignant tumors. Several driver mutations have been identified in the development of GIST, with the most frequent being found in the tyrosine kinase receptor c-kit (c-KIT). Other relevant mutations have been observed in the Platelet Derived Growth Factor Receptor Alpha (PDGFR-α). Their diagnosis is usually based on the expression of CD117 (90–95%), a membrane-bound receptor of tyrosine kinase, and CD34 (70–80%) [7].

GISTs commonly co-exist with other primary tumors, which can involve either the GI tract or other extra-GI sites. Synchronous occurrence of GISTs with other malignancies varies from 4.5% to 33%, with the most frequent localization of GIST- associated malignancies in the GI tract, the urogenital and female genital tract [8]. The general probability of a second malignancy during one’s lifetime is estimated to be 2–17%. In comparison, the rate of a second malignancy in GIST patients is higher than expected. GISTs with synchronous malignancy of the GI tract are described as ranging from 10% to 35%, with the majority of them being adenocarcinomas [1, 9–11, 12].

It is not yet clear if a causal association exists for the concomitant occurrence of GIST with other malignancies, or if this is merely coincidence. Furthermore, its clinical importance has yet to be determined. The occurrence of GIST with other synchronous primary malignancies has primarily been described in the literature through case reports and rarely as case series, which is not sufficient for proving any association between these two entities.

## RESULTS

### Patients’ tumor characteristics

A total of sixty-nine patients with GISTs were included in our study; thirty-five patients (51%) were male and thirty-four (49%) were female. The median age at time of diagnosis was 66 years (range 28–88). In 37 cases (53.6%) GISTs were located in the stomach and in 11 cases (15.9%) they were located in the small intestine, there were 5 cases (7.2%) where GISTs were located in the duodenum, 4 cases (5.8%) with GISTs in the colon and sigmoid, 3 cases (4.3%) with GISTs in the mesentery, 2 cases (2.9%) with GISTs in the pelvis, and in 7 cases (10.1%) GISTs were found in other locations (omentum, ligamentum teres, presacral space, gallbladder, liver, pancreas and muscle-soft tissues). The median tumor size was 6.3 cm. Risk of tumor recurrence was defined using the tumor size, mitotic rate and location. A total of 29 (42%) were classified as high risk, 12 (17%) as moderate risk and 28 (41%) as low risk.

In the immunohistochemical analysis of all patients with GIST, c-KIT (CD-117) was positive in 53/69 cases (77%), CD-34 was positive in 34/69 cases (49%), SMA was positive in 10/69 cases (14%), S-100 was positive in 15/69 cases (22%), desmin was positive in 4/69 cases (6%), L1CAM was positive in only 1/69 cases (1.5%), DOG1 was positive in 13/69 cases (19%), and vimentin was positive in 17/69 cases (25%).

The prevalence of other primary malignancies in patients with a GIST was calculated at 10.1% (7/69). In all 7 patients, GISTs were an incidental finding during the preoperative staging or intraoperatively for other primary tumors. The presence of tarry stools was the leading cause of admission (4/7), followed by hematemesis (2/7), and only one patient was admitted with intestinal obstruction (Table 1). Five (71%) of the synchronous GISTs were located in the stomach and two (29%) were located in the small intestine. Moreover, five of the second primary tumors (71%) were adenocarcinomas and two (29%) were liposarcomas; four (57%) of these were located in the stomach, two (29%) in the colon and one (14%) in the retroperitoneal (Table 2). The median tumor size of synchronous GISTs was 3 cm. As it concerns the risk stratification, only one (14%) was classified as high risk (14%) and one (14%) as moderate risk, while five (72%) were classified as low risk (Table 3).

**Table 1:** Patients characteristics with GIST and other synchronous primary tumors

Patient number	Age	Gender	Location of Synchronous Primary Tumor	Cause of Admission
1	82	Male	Stomach	Melena
				Anemia
2	55	Male	Stomach	Melena
				Anemia
				Hematemesis
3	73	Female	Stomach	Melena
				Hematemesis
4	84	Male	Cecum	Anemia
5	56	Male	Retroperitoneal	Hematemesis
6	75	Male	Stomach	Melena
7	67	Male	Transverse colon	Intestinal Obstruction

**Table 2:** Pathological characteristics of synchronous GIST and other primary tumors

Patient number	GIST		Synchronous Primary Tumor	
	Location	Size (cm)	Type	Location
1	Stomach	1.1	Liposarcoma	Stomach
2	Small intestine	4.5	Adenocarcinoma	Stomach
3	Small intestine	3	Adenocarcinoma	Stomach
4	Stomach	4.5	Adenocarcinoma	Cecum
5	Stomach	10	Liposarcoma	Retroperitoneal
6	Stomach	2.2	Adenocarcinoma	Stomach
7	Stomach	2.1	Adenocarcinoma	Transverse colon

**Table 3:** Histopathologic features of the synchronous GISTs and follow-up

Patient number	Mitotic Index (x/50 hpf)	Risk Group	C-KIT	CD-34	SMA	S-100	DOG1	Vimentin	TKI Therapy	Follow-Up (Months)
1	1–2	Low	+	+	–	+	+	–	No	Disease Free (48)
2	1–2	Low	+	+	–	–	+	–	No	Death (48)
3	2–3	Low	+	+	+	–	–	+	No	Disease Free (48)
4	< 2	Low	+	+	–	+	+	–	No	Death (60)
5	4–6	High	+	+	–	+	–	+	No	Death (13)
6	> 5	Moderate	+	+	–	–	+	–	No	Recurrence (36)
7	< 5	Low	+	+	+	+	+	–	No	Recurrence (60)

(abbreviation: TKI- Tyrosine Kinase Inhibitor).

The immunohistochemical analysis of patients with synchronous GIST and another primary tumor C-KIT was positive in 7/7 cases (100%), CD-34 in 7/7 cases (100%), SMA was positive in 2/7 cases (29%), S-100 was positive in 4/7 cases (57%), DOG1 was positive in 5/7 cases (71%), DOG1 was positive in 5/7 cases (71%) and Vimentin was positive 2/7 cases (29%) (Table 3).

Data from follow up of the 69 patients who underwent surgical treatment for a single GIST have reported that 33 patients (48%) received Imatinib as adjuvant chemotherapy, 2 patients (3%) received Sunitinib, 1 (1%) patient switched to Sunitinib after adverse effects from receiving Imatinib, and 33 patients (48%) did not receive any adjuvant therapy. All patients receiving adjuvant treatment were stratified as either intermediate or high risk.

All patients with synchronous malignancy underwent radical surgery for the other primary tumor and complete resection (R0) for the GIST simultaneously. GISTs were removed en-bloc with the other tumor, or a separate resection was performed when the GIST was located distal to the other mass. All patients had a close oncological follow-up for years. Of the seven patients with concurrent GIST and another primary tumor, 4 received adjuvant chemotherapy and/or radiotherapy targeting the concurrent malignancy and not the GIST. None of the patients in the synchronous group received any therapy with Tyrosine Kinase Inhibitors (TKIs). Two patients remain disease-free 48 months after the surgical treatment, two patients experienced local recurrence after 36 and 60 months, respectively, and three patients died in 13, 48 and 60 months, respectively (Table 3).

### Statistical analysis

**Figure 1:**
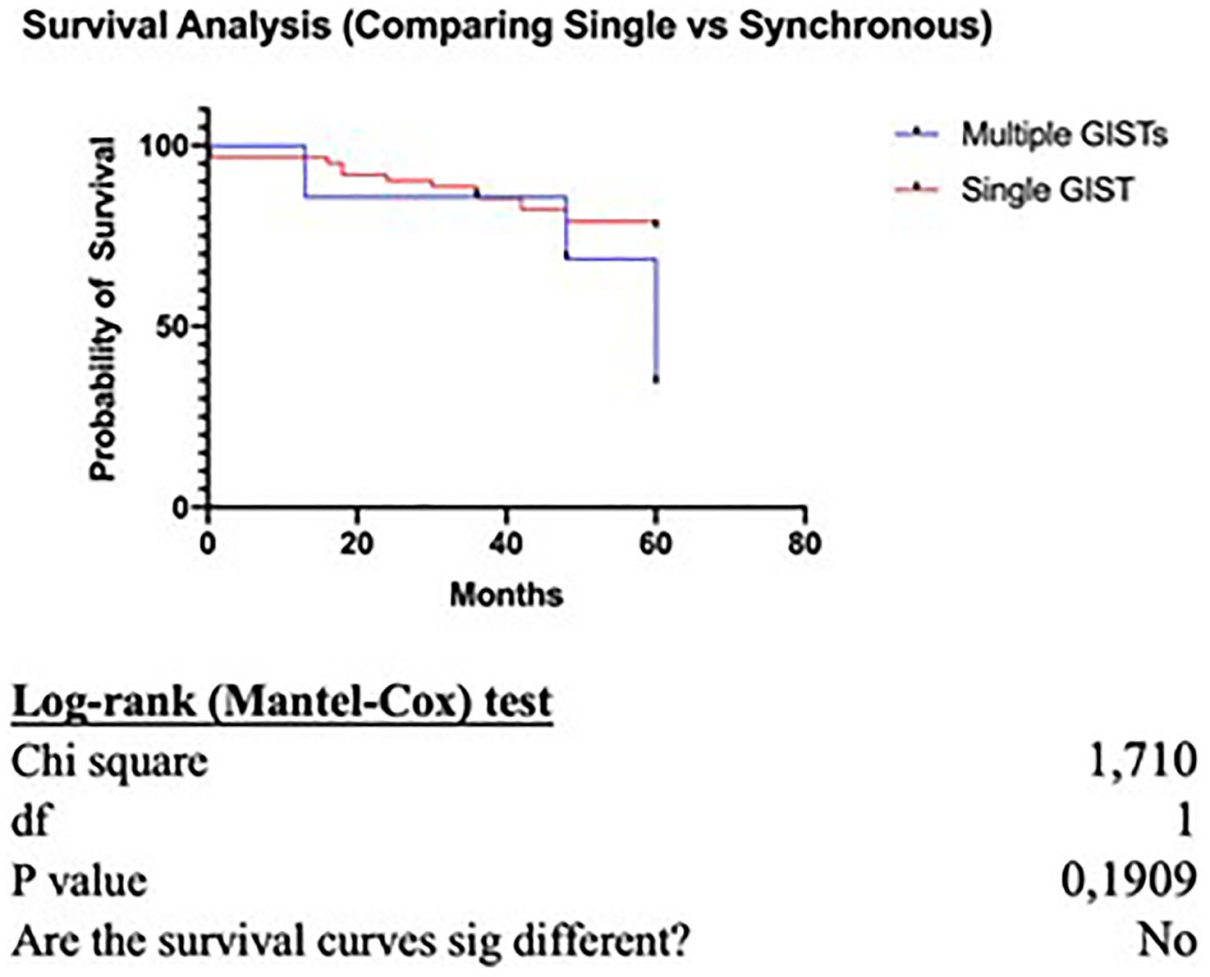
Survival analysis.

**Figure 2:**
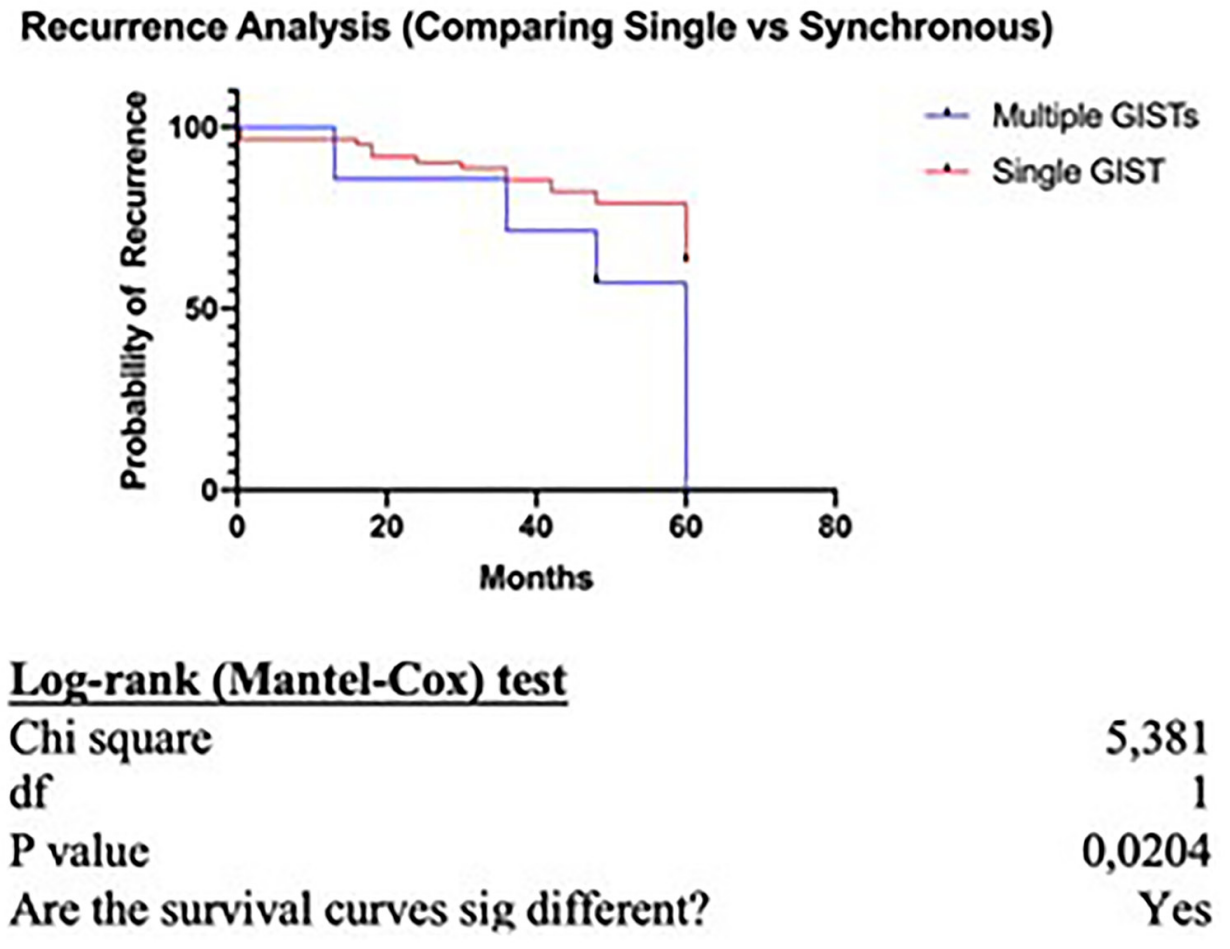
Reccurence analysis.

Survival analysis comparing the overall survival of patients with single GIST versus patients with concurrent GIST and another primary tumor, has shown no statistically significant difference between the two groups (*p* = 0.19) (Figure 1). However, when comparing the recurrence rate, patients with synchronous GISTs and another primary tumor have a statistically significant increased possibility for recurrence (*p* = 0.02) (Figure 2).

**Figure 3:**
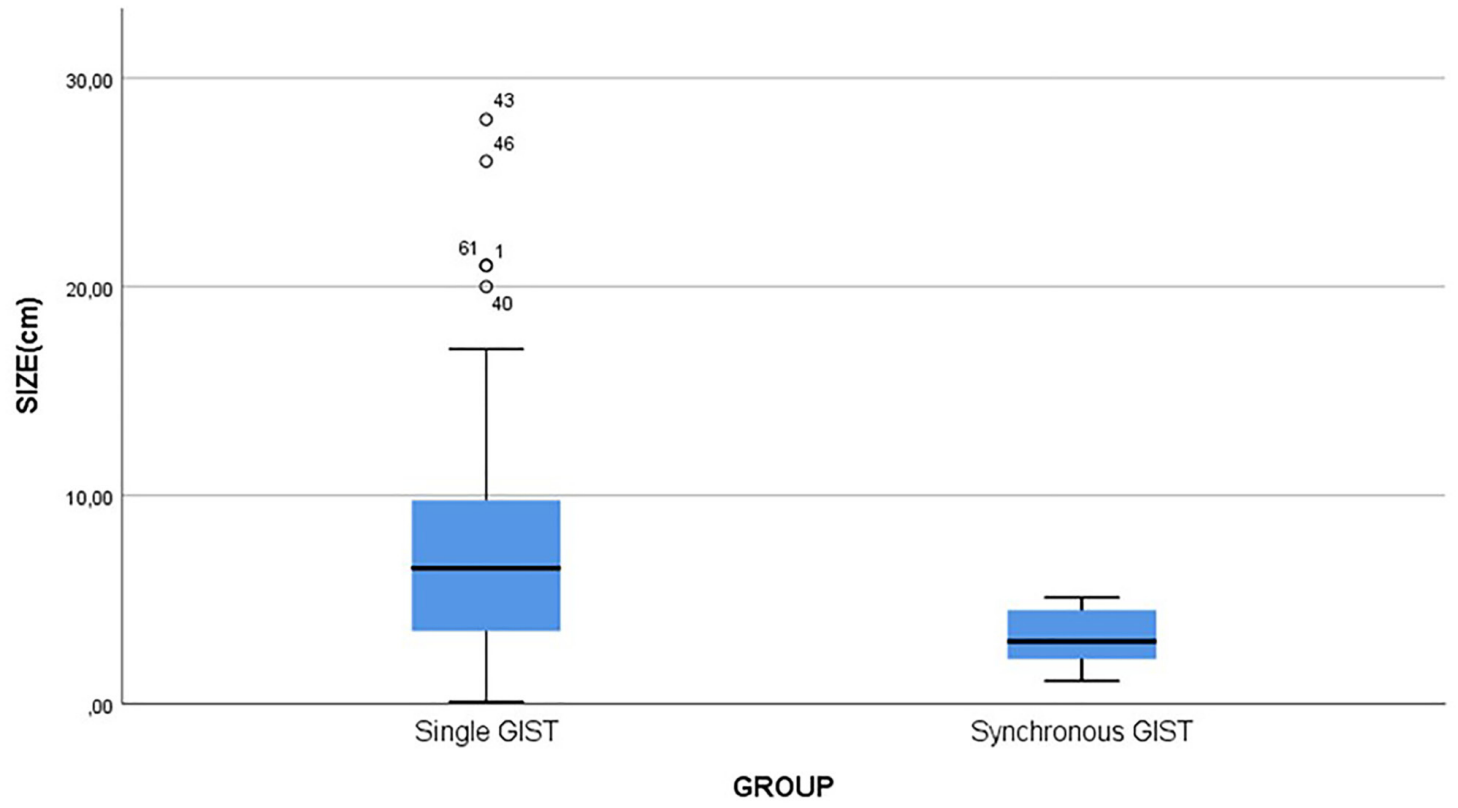
Comparison of tumor size.

**Figure 4:**
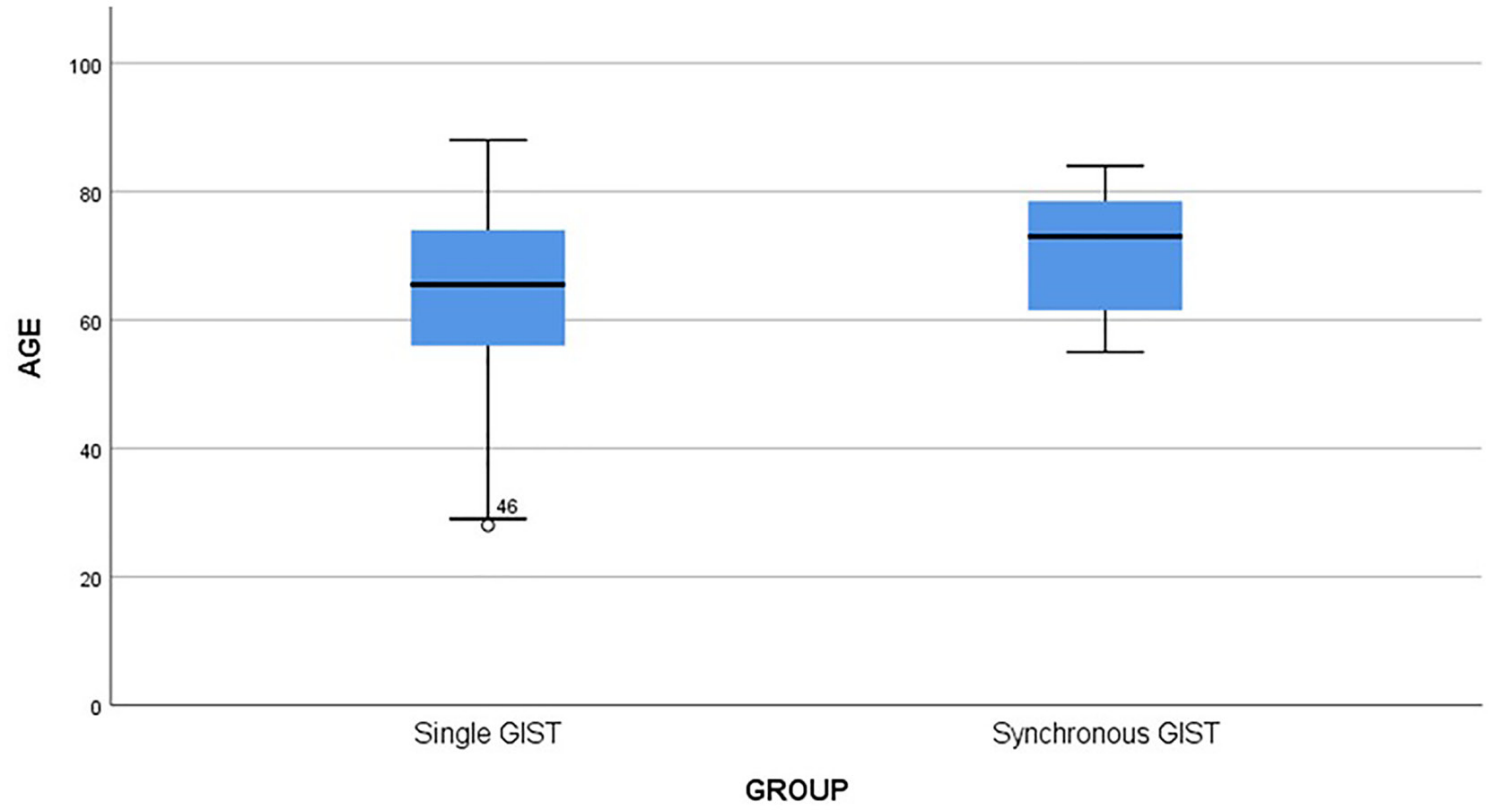
Age comparison.

When comparing the size of GISTs between the two groups (a single GIST or synchronous GIST with another primary tumor), statistical analysis has shown that patients with single GIST have larger tumors than patients with synchronous GISTs (*p* = 0.02) (Figure 3). The median age in the group of patients with synchronous malignancies was higher (median = 73 years) compared to patients with a single GIST (median = 65.5 years); however, the difference was not statistically significant (*p* = 0.27) (Figure 4). In addition, the proportion of high-risk GISTs and the gender were not statistically different between the two groups (*p* = 0.226 for high-risk and *p* = 0.106 for gender).

## DISCUSSION

According to a recent meta-analysis published in 2019 [14], the prevalence of second primary tumors in patients with a diagnosis of GIST is 20.4%, with the most common anatomic locations of tumor development being the gastrointestinal and genitourinary tracts. Furthermore, the rate of synchronous second neoplasia is believed to be between 4.5–33% [8, 13, 14].

Currently in the literature, three theories mainly describe the pathogenetic mechanisms underlying the occurrence of synchronous GIST-associated tumors. The model most commonly discussed in the literature is that of a non-causal relationship between GIST and other malignancies, suggesting that two or more tumors occur together incidentally [15]. This rationale developed based on studies reporting that the real annual incidence of GISTs is much higher than the customarily mentioned 1.5/100,000 [16]. In fact, Kawanowa et al. [17] report that the incidence of asymptomatic microscopic GIST in stomachs resected for stomach carcinoma is 35%, and Nilsson et al. [18] report that incidental GIST is found in 0.2% of all autopsies.

In contrast to the incidental, non-causal relationship model, some studies have suggested that exposure to potential carcinogens can trigger oncogenetic pathways in both epithelial and mesenchymal cells [19, 20]. Namely, Cohen et al. [21] showed that the combined exposure to nitrosoguanidine and acetylsalicylic acid induced the development of simultaneous gastric cancer and leiomyosarcoma in rat models. As a consequence, they speculated that carcinogens may interact with tissues adjacent to one another resulting in the occurrence of histologically different tumors in the same organ. Finally, common genetic mutations, which are present in both epithelial and stromal cells, are hypothesized to be the cause of the synchronous tumor occurrence. Presently there is no conclusive evidence to support this theory [14, 22].

According to our results and in concordance with the current literature, the most common GIST- associated neoplasms are reported to be adenocarcinomas of the gastrointestinal tract, comprising up to 38% of all the second primary tumors found in patients with GISTs [14]. In synchronous cases, GISTs are usually found incidentally during the surgical operation or the histopathologic examination of the resected tissue [23, 24]. Interestingly, GISTs are not the only type of mesenchymal tumors that have been reported to occur concurrently with adenocarcinomas. Cases of gastric adenocarcinomas alongside leiomyosarcomas [25], rhabdomyosarcomas [25], or tumors with sarcomatous stromal components [26] have been previously described.

GIST-associated sarcomas are rarely reported in the literature considering that soft tissue tumors comprise only 0.5–3% of all GIST-associated neoplasms [12, 14]. Cases of synchronous, GIST-associated sarcomas are even less frequently reported and they are usually liposarcomas [27], angiosarcomas [28], angiomyoliposarcomas [24], and leiomyosarcomas [29, 30]. Intriguingly, synchronous sarcoma-associated sarcomas are also rare clinical entities. Some of the combinations reported are extremity liposarcoma with retroperitoneal leiomyosarcoma [27], extremity liposarcoma with solitary fibrous tumor [30], and uterine leiomyosarcoma with retroperitoneal liposarcoma [31]. In our study, we report two extremely rare cases of patients with GIST and synchronous liposarcoma. The GIST in both cases was located in the stomach, while the synchronous liposarcoma in one patient was also located in the stomach and in the second one in the retroperitoneum.

In our case series the mean GIST size was statistically significantly higher in patients with GIST only. On the other hand, tumors with smaller size and very low or low risk of malignant potential were found in patients with synchronous malignancy, supporting the theory that the GIST itself in these patients (synchronous group) has only little or no effect at all on the overall survival and prognosis. Even if our survival analysis failed to show a statistically significant difference between the two groups (*p* = 0.19) when comparing the recurrence rate, patients with synchronous disease had an increased possibility for recurrent disease (*p* = 0.02) resulting in worse prognosis and a more aggressive course.

Another interesting observation relates to the markers’ positivity in the immunohistochemical analysis. The most important markers for defining GISTs are CD117 (c-kit protein) and CD34 (hematopoietic cell progenitor antigen). Thus, although the vast majority of GISTs are positive for CD117 (> 95%) and/or CD34 (40%), the simultaneous detection of both CD117 and CD34 is not a feature observed in the majority of the patients with GISTs. Interestingly, in our case series, all seven patients with synchronous malignancy expressed both CD117 and CD34 comprising a possible explanation of the association of the synchronous malignancies.

### Strengths and limitations of the study design

The present study compared and associated the overall survival, the recurrence rate, the tumor size in patients with a single GIST and in patients with synchronous GIST and another primary tumor. However, the data were collected retrospectively depending on the availability and accuracy of the data records, and information bias may have occurred. Due to the limited number of patients in the synchronous GIST group, the results of the statistical analysis should be interpreted cautiously.

## MATERIALS AND METHODS

### Patients

A retrospective study was conducted using medical and pathological records from sixty-nine patients who underwent surgical treatment for GIST in a single university’s surgical department between 2011 and 2019. Seven cases of GISTs accompanying a synchronous primary tumor were identified and included in the study.

### Ethical considerations

Considering the retrospective study design based primarily on medical records and pathological results, as well as the anonymity of patients in the statistical analysis, ethical committee approval was not obtained. Experimental therapeutic protocols are not applicable in this study. All data were analyzed anonymously using code numbers with respect to the patient’s privacy, and collected in the context of routine diagnostic and therapeutic procedures. Nevertheless, informed verbal consent was obtained from the subjects.

### Histological and immunohistochemistry analyses

Specimens were fixed in 10% formaldehyde and processed routinely for paraffin embedding. Sections 3-mm thick were stained with hematoxylin-eosin. Mitoses were counted in 50 high-power fields.

Immunohistochemistry analysis using a panel of commercially available antibodies directly against the following antibodies (c-kit, CD-34, SMA, S-100, DOG1 and Vimentin) were performed in order to define and corroborate the diagnosis.

### Statistical analysis

Statistical calculations were performed in SPSS v.25 (IBM Chicago, IL, USA). As the data did not follow a normal distribution (verified with a Shapiro-Wilk test), the non-parametric Mann-Whitney *U* test was used to determine if a statistically significant difference existed in tumor size and age between the two groups. A chi-square test was used to identify if a statistically significant difference existed between the two groups, as it concerned the proportions of high-risk GIST and gender. A *p*-value < 0.05 was considered statistical significant Recurrence and survival analysis were performed using Kaplan-Meier curves, and the curves were compared using the Log-rank (Mantel-Cox) test.

## CONCLUSIONS

In conclusion, the synchronous occurrence of GIST and other primary tumors is more common than it has been considered in the past decades, with the majority of them being found incidentally during surgery performed for another malignancy. Even though coincidence may be the answer, the hypothesis of gene mutations or the same carcinogenic agent resulting in two tumors of different origin cannot be excluded. Further research based on large populations and cancer registries, as well as on molecular mechanisms, and probably even animal experiments, are required to elucidate any association between GISTs and other malignancies. In addition, despite the fact that the prognosis of these patients is mainly determined by the other malignancy and not by the GIST, all physicians must be aware of possible synchronicity, recognize the gastrointestinal stromal tumor and apply the proper therapeutic protocol to the patient.

### Ethical approval

Ethical committee approval was not obtained. Nevertheless, informed verbal consent from the subjects was obtained.
